# Hematopoietic-restricted Ptpn11E76K reveals indolent MPN progression in mice

**DOI:** 10.18632/oncotarget.25073

**Published:** 2018-04-24

**Authors:** Stefan P. Tarnawsky, Wen-Mei Yu, Cheng-Kui Qu, Rebecca J. Chan, Mervin C. Yoder

**Affiliations:** ^1^ Department of Biochemistry and Molecular Biology, Indiana University School of Medicine, Indianapolis, IN, USA; ^2^ Department of Pediatrics, Emory University School of Medicine, Atlanta, GA, USA; ^3^ Department of Pediatrics, Herman B. Wells Center for Pediatric Research, Indiana University School of Medicine, Indianapolis, IN, USA; ^4^ Department of Medical and Molecular Genetics, Indiana University School of Medicine, Indianapolis, IN, USA

**Keywords:** JMML, MPN, PTPN11, lineage tracing

## Abstract

Juvenile Myelomonocytic Leukemia (JMML) is a pediatric myeloproliferative neoplasm (MPN) that has a poor prognosis. Somatic mutations in Ptpn11 are the most frequent cause of JMML and they commonly occur *in utero*. Animal models of mutant Ptpn11 have probed the signaling pathways that contribute to JMML. However, existing models may inappropriately exacerbate MPN features by relying on non-hematopoietic-restricted Cre-loxP strains or transplantations into irradiated recipients. In this study we generate hematopoietic-restricted models of Ptpn11E76K-mediated disease using Csf1r-MCM and Flt3Cre. We show that these animals have indolent MPN progression despite robust GM-CSF hypersensitivity and Ras-Erk hyperactivation. Rather, the dominant pathology is pronounced thrombocytopenia with expanded extramedullary hematopoiesis. Furthermore, we demonstrate that the timing of tamoxifen administration in Csf1r-MCM mice can specifically induce recombinase activity in either fetal or adult hematopoietic progenitors. We take advantage of this technique to show more rapid monocytosis following Ptpn11E76K expression in fetal progenitors compared with adult progenitors. Finally, we demonstrate that Ptpn11E76K results in the progressive reduction of T cells, most notably of CD4+ and naïve T cells. This corresponds to an increased frequency of T cell progenitors in the thymus and may help explain the occasional emergence of T-cell leukemias in JMML patients. Overall, our study is the first to describe the consequences of hematopoietic-restricted Ptpn11E76K expression in the absence of irradiation. Our techniques can be readily adapted by other researchers studying somatically-acquired blood disorders.

## INTRODUCTION

Juvenile Myelomonocytic Leukemia is a pediatric myeloproliferative neoplasm (MPN) that has limited therapeutic options and a poor prognosis. The majority of JMML cases are the result of somatic mutations in Ras signaling mediators. Hematopoietic-restricted Ptpn11 mutations are found in 10–37% of JMML patients and are the most frequent polymorphism in this disease [1–3]. Mutant Ptpn11 evokes hyperactive Ras signaling among hematopoietic progenitors that causes growth hypersensitivity in the presence of the cytokine GM-CSF [4]. Patients develop monocytosis, anemia, thrombocytopenia, and hepatosplenomegaly. The only definitive treatment for JMML is an allogeneic hematopoietic stem cell (HSC) transplant. Nevertheless, within 5 years of transplant 33% of patients will relapse and their overall survival is 52% [5].

Historically, the majority of JMML-initiating mutations were thought to be mutually exclusive [6, 7]. Recent evidence, however, has identified that many patients have cooperative mutations in Ras-mediated pathways [1–3]. Nevertheless, a single mutation is sufficient to give rise to JMML. As such, animal models of JMML have been readily generated that utilize transplantation of mutation-expressing hematopoietic progenitors or Cre-loxP conditional gene targeting [8–14]. These approaches, however, have notable drawbacks. First, post-transplant hematopoiesis is markedly different from unperturbed hematopoiesis. Transplanted progenitors have altered lifespans, biased differentiation potentials, and frequently give rise to different disease manifestations when compared with progenitors in non-transplanted hosts [15–17]. Second, whereas somatic JMML mutations are restricted to hematopoietic cells, the majority of Cre stains used in the study of JMML have promiscuous tissue activity. In addition to their activity in the hematopoietic lineage, VavCre is also expressed in endothelial cells, LysMcre is also expressed in lung stromal cells, and Mx1Cre is also expressed in endothelial cells, BM mesenchymal cells, and in the GI tract [18, 19]. Hyperactive Ras expression in these non-hematopoietic tissues exacerbates features of MPN and leads to solid-tissue tumors [18–20]. There is a need, therefore, to develop animal models of JMML that can more accurately recapitulate the pathogenesis of the childhood disease.

The origin of JMML is tightly associated with development. The median age at diagnosis is less than 2 years and the disease bears distinct features of fetal hematopoiesis including an overabundance of fetal hemoglobin and a unique gene expression signature [21, 22]. Furthermore, fetal progenitors have greater myeloid colony forming potential compared to adult progenitors, suggesting a mechanism whereby they may rapidly give rise to a myeloid disease [23]. Finally, retrospective testing of patient tissues collected at birth suggests that the majority of somatic JMML-initiating mutations occur prenatally [3, 24, 25]. We have recently shown that *in utero* expression of the JMML-initiating KrasG12D mutation results in a remarkably faithful disease model [26]. However, the relative contribution of fetal vs. adult progenitors to JMML remains unknown. We therefore sought to develop a strategy to induce hematopoietic-restricted Ptpn11E76K expression in fetal or adult progenitors in the absence of myeloablation. We hypothesized that fetal progenitors expressing Ptpn11E76K would demonstrate faithful disease progression and reveal previously underappreciated features of this disease.

In this study we generated hematopoietic-restricted models of Ptpn11E76K that do not rely on transplantation. We performed serial peripheral blood analysis to determine the progression of the disease in a non-myeloablated host. Finally, we developed a strategy that induces Ptpn11E76K expression in either fetal or adult hematopoietic progenitors and we compared MPN outcomes following *in utero* vs. postnatal oncogene expression. We have thereby generated faithful representations of JMML pathophysiology that can serve as pre-clinical models. Moreover, we have identified a previously unappreciated paucity of T cells in the setting of mutant Ptpn11E76K. This findings corresponds with altered T cell development in the thymus of mutant mice and may help explain reports of T-ALL emergence in JMML patients.

## RESULTS

**Figure 1:**
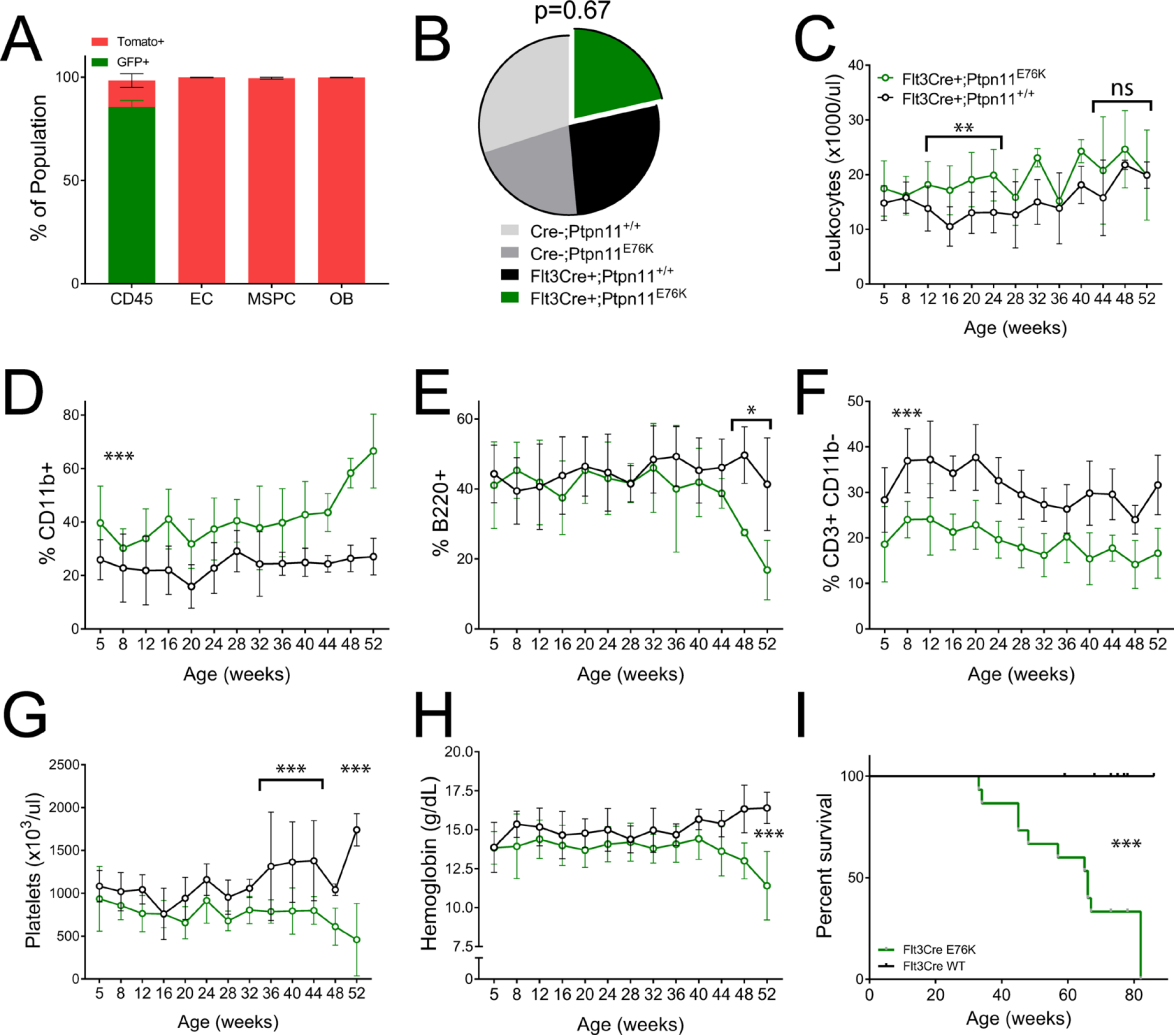
Flt3Cre+ Ptpn11E76K mice acquire an indolent MPN (**A**) Cre activity as measured by GFP expression in BM subsets of 4 week old Flt3Cre+ Rosa26^mTomato/mGFP^ mice (*n =* 3). (**B**) Birth ratios of Flt3Cre+ x Ptpn11^E76K/+^ matings with chi-squared analysis (**C–H**) Serial peripheral blood analysis of leukocytes frequency and lineage distribution, platelet count and hemoglobin abundance in mutants (*n =* 10) and littermate controls (*n =* 12). Percentages represent each lineage’s proportion among all mononuclear cells. *P*-values represent *t*-tests between mutants and controls. (**I**) Kaplan–Meier survival analysis of mutants (*n =* 15) and littermates (*n =* 18). EC, endothelial cells. MSPC, mesenchymal stem/progenitor cell. OB, osteoblast.

We confirmed the hematopoietic-restricted expression of Flt3Cre using the Rosa26mTomato/mGFP (mTmG) model [27]. Therein, cells that express Cre undergo an irreversible switch from Tomato to GFP expression. We measured the frequency of GFP+ cells among BM stromal populations in 4 week old mice using flow cytometry. As expected, the majority of CD45+ BM cells were GFP+, indicating robust Cre recombinase activity in this population (Figure 1A). In contrast, endothelial cells (Ter119- CD45- CD31+ Sca1+), osteoblasts (Ter119- CD45- CD31- CD140a+ Sca1-) and mesenchymal progenitor cells (Ter119- CD45- CD31- CD140a+ Sca1+) were Tomato+. This confirms that Flt3Cre is not active in BM stromal progenitors and strongly suggests that this Cre is hematopoietic-restricted. We therefore proceeded to mate Flt3Cre+; Rosa26mTmG/mTmG mice and Ptpn11E76K mice to generate Flt3Cre+;Rosa26mTmG/+; Ptpn11E76K/+ (Flt3Cre+; E76K) mutants and Flt3Cre+;Rosa26mTmG/+;Ptpn11+/+ (Flt3Cre+; WT) controls.

Flt3Cre+;E76K mutants were born at expected Mendelian ratio and had markedly myeloid-biased peripheral leukocytes beginning at 5weeks of age compared to littermate controls (Figure 1B, 1D). There was a concomitant decrease in T cells without changes in the frequency of B cells. The relative frequency of peripheral myeloid cells, B cells, and T cells did not change between 5–48 weeks of age, at which time there was a pronounced increase in myeloid cells and a concomitant decrease in B cells (Figure 1E, 1F). The CD4:CD8 ratio among T cells in mutants was equal to that in controls until 32 weeks of age. Thereafter, mutants show a preferential decrease in CD4+ T-cells (Supplementary Figure 1). Whereas mutant mice also had a pronounced thrombocytopenia and progressive anemia, there was no clear trend towards leukocytosis (Figure 1C, 1G, 1H). This suggested that Flt3Cre+; E76K mice would have prolonged survival compared with previous mouse models that expressed this oncogene. Indeed, the median survival of Flt3Cre+;E76K mice was 66 weeks of age, compared with historic median survivals of 36 weeks for LysMCre+;E76K mice and 28 weeks for Mx1Cre+;E76K mice, respectively [11] (Figure 1I). These results suggest that in the absence of stromal cell expression the MPN initiated by Ptpn11E76K demonstrates indolent progression.

Flt3Cre is active in fetal multipotent progenitors beginning *in utero* at around E10.5. However, Flt3Cre activity will continue to emerge in MPPs after 4 weeks of age, which marks the end of the transition from fetal to adult hematopoiesis [28]. As such, this Cre strain cannot discern the distinct contribution of fetal and adult hematopoietic programs to Ptpn11E76K-mediated disease in aged mice. Given that the majority of JMML patients have a fetal-like gene expression signature, we set out to identify a Cre strain that could uniquely activate Ptpn11E76K expression in either the fetal or the adult hematopoietic programs.

**Figure 2:**
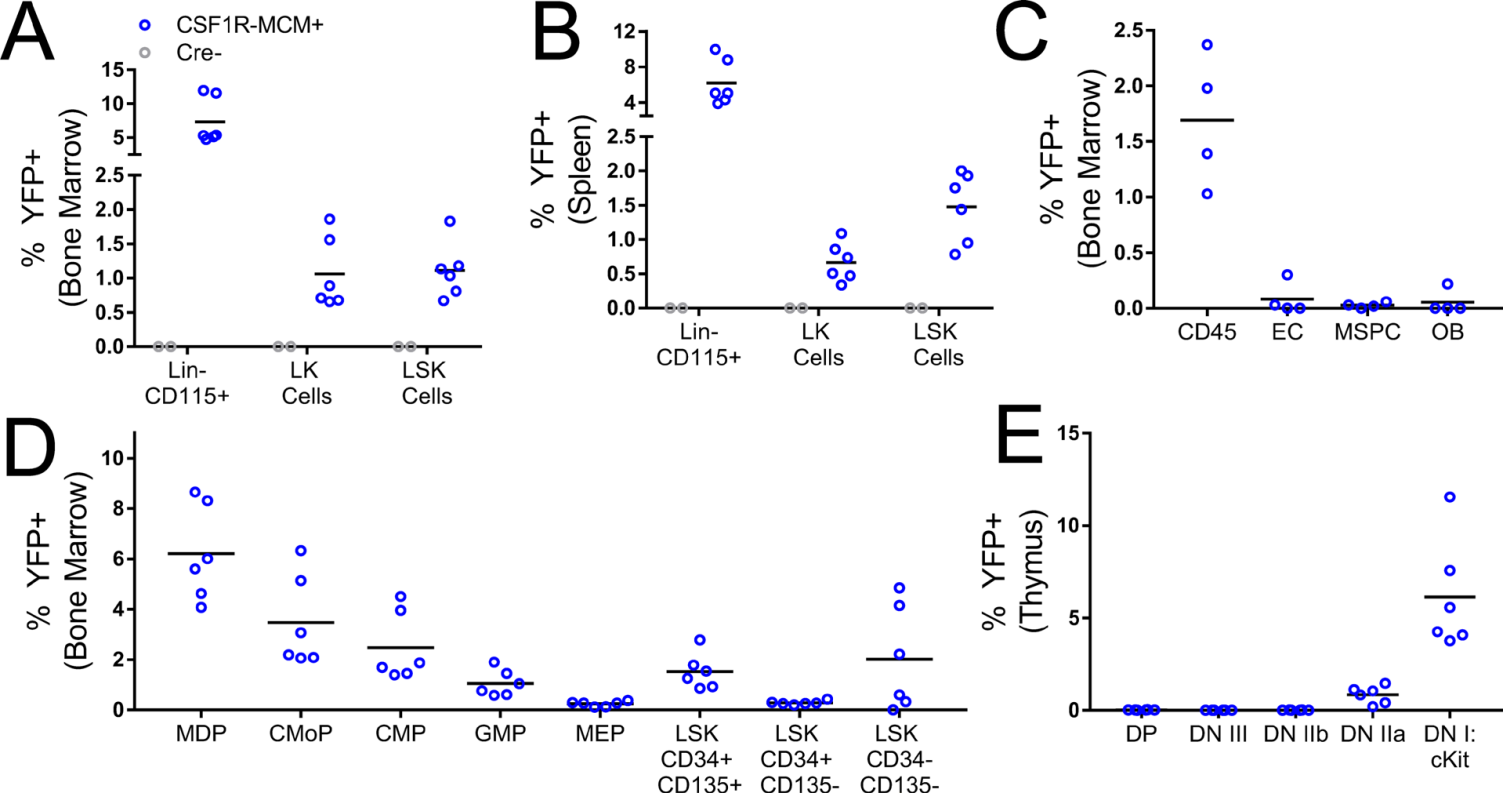
Csf1r MER-Cre-MER activity in hematopoietic progenitors and BM stromal cells Csf1r-MCM+ Rosa26^YFP^ animals were analyzed 1week after injections with tamoxifen. (**A–B**) Frequency of YFP+ BM and splenic progenitors. (**C**) Frequency of YFP+ BM stromal populations. (**D**) Analysis of YFP+ BM progenitors in Csf1r-MCM+ Rosa26^YFP^ animals. (**E**) Frequency of YFP+ thymus progenitors. LK, Lin- cKit+. LSK, Lin- Sca1+ cKit+. EC, endothelial cells. MSPC, mesenchymal stem/progenitor cell. OB, osteoblast. MDP, myeloid-dendritic progenitor. CMoP, common monocyte progenitor. CMP, common myeloid progenitor, GMP, granulocyte-macrophage progenitor. MEP, megakaryocyte-erythrocyte progenitor. DP, CD4 CD8 double positive. DN, double negative.

To this end, we characterized the fluorescence expression pattern in Csf1r Mer-Cre-Mer;Rosa26YFP mice (Figure 2). In this model, recombinase activity requires two simultaneous signals. First, cells must express Csf1r. Second, they must be exposed to tamoxifen, which will bind the MER domains and permit the nuclear localization of the Cre enzyme. We reasoned that by timing the dose of tamoxifen we would be able to activate recombinase activity in either fetal or adult hematopoietic progenitors. We injected 4 week old Csf1r-MCM+; Rosa26YFP mice with 3 doses of 75 µg/g tamoxifen. 7 days later, we measured the expression of YFP in hematopoietic progenitor subsets. Whereas no YFP+ cells were observed in Cre- littermate controls, Csf1r-MCM+ mice consistently had YFP+ LK and LSK cells in the BM and spleen, including LSK CD34- CD135- HSCs (Figure 2A, 2B, 2D). Furthermore, we observed YFP+ early thymic progenitors (CD4- CD8- CD25- CD44+ cKit+) in Csf1r-MCM+ mice (Figure 2E). Importantly, very few BM stromal cells – such as endothelial cells, osteoblasts, and mesenchymal progenitors – were YFP+ (Figure 2C). These findings confirm that exposure of Csf1r-MCM+ mice to tamoxifen at 4 weeks of age would consistently target hematopoietic progenitors of the adult phase.

To determine whether a similar strategy could be used to target fetal hematopoietic progenitors, we performed timed matings between Csf1r-MCM+ studs and Rosa26YFP/YFP dams. At E14.5, pregnant dams were injected with 75 µg/g 4-hydroxy tamoxifen. 48 hours later, hematopoietic progenitors were isolated from the livers of fetuses and YFP expression was measured using flow cytometry. We observed consistent YFP+ expression in LSK cells, including LSK CD150+ CD48- fetal HSCs, in Csf1r-MCM+ embryos (Supplementary Figure 2). No YFP+ cells were observed in Cre- littermates. These findings suggest that timed exposure of Csf1r-MCM mice to tamoxifen would permit specific and consistent activation of recombinase activity in either fetal or adult hematopoietic progenitors. Additionally, it suggested that cells expressing mutant Ptpn11 could be identified via the expression of YFP.

**Figure 3:**
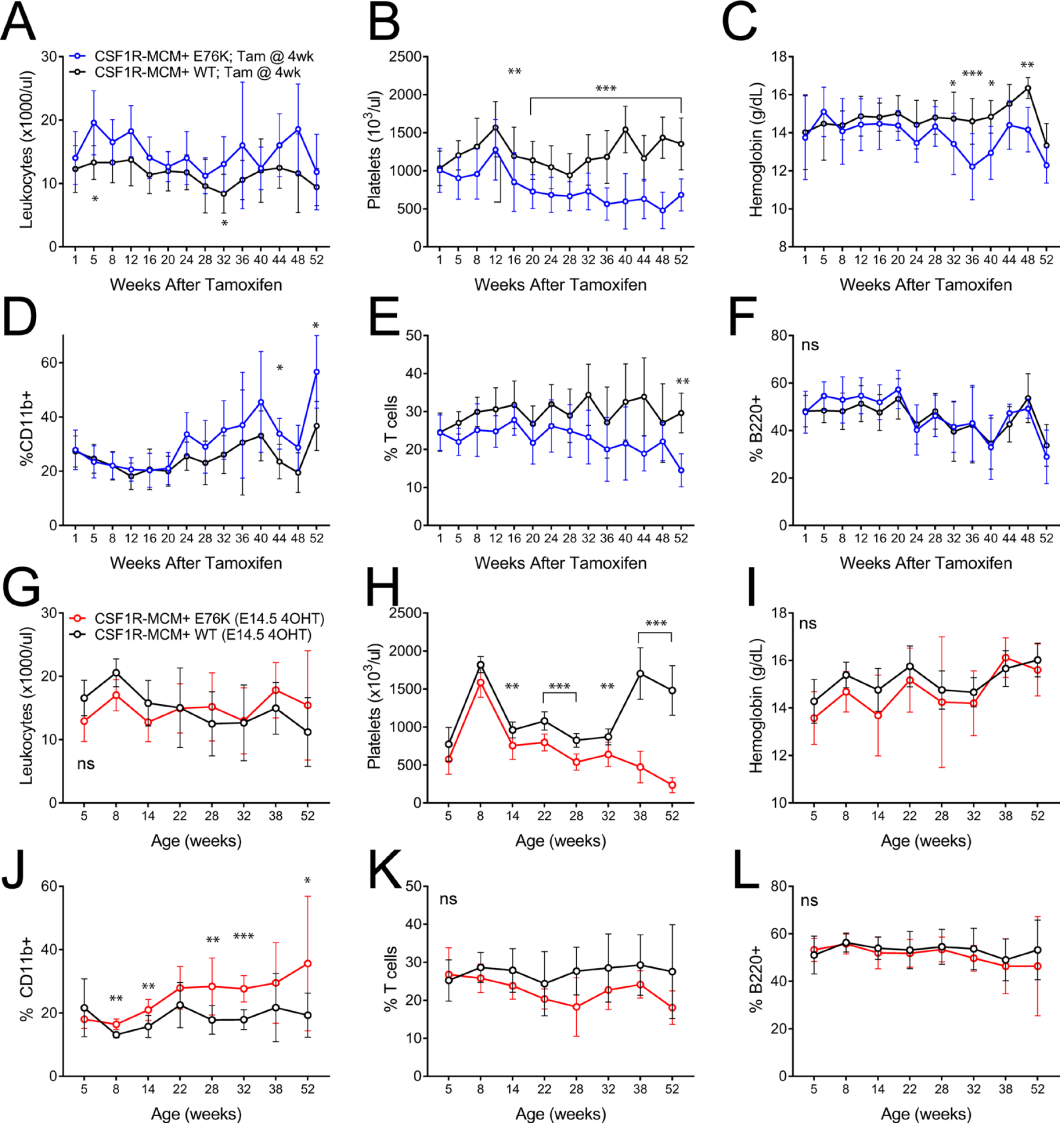
Peripheral blood comparison of Csf1r-MCM+ Ptpn11E76K fetal and adult cohorts (**A–F**) Animals in the adult cohort were exposed to tamoxifen at 4 weeks (*n =* 12 mutants and 16 WT) (**G–L**) Animals in the fetal cohort were exposed to tamoxifen at E14.5. (*n =* 7 mutants and 10 WT) A,G) Leukocyte counts. (B, H) Frequency of YFP+ cells. (C, I) platelet counts. (D, J) Frequency of myeloid cells. (E, K) Frequency of T-cells. (F, L) Frequency of B cells. Percentages represent each lineage’s proportion among all mononuclear cells. *P* values represent *t*-tests comparing mutants and controls.

We proceeded to generate fetal and adult cohorts of Csf1r-MCM+;Rosa26YFP; Ptpn11E76K (Csf1r-MCM+;E76K) animals that had been exposed to tamoxifen at either E14.5 or at 4 weeks of age, respectively. We performed peripheral blood analysis of mutant animals and Csf1r-MCM+;RosaYFP;Ptpn11+/+ (Csf1r-MCM+;WT) littermate controls (Figure 3). Mutant animals in the adult cohort did not demonstrate consistent leukocytosis (Figure 3A). Whereas the animals did acquire thrombocytopenia, they were only moderately anemic (Figure 3B, 3C). Surprisingly, adult-cohort mutants showed markedly delayed monocytosis beginning 44 weeks after tamoxifen, as measured by %CD11b+ cells. This coincided with a decline in the frequency of T cells—as measured by CD3+, CD4+, or CD8+ cells—without a change in B220+ B cell frequency (Figure 3D–3F, Supplementary Figures 3, 4).

We compared these findings to Csf1r-MCM+;E76K mutants whose oncogene became active *in utero* at E14.5 (Figure 3G–3L). Mutants in this fetal cohort showed monocytosis beginning at 8 weeks of age—considerably sooner than mutants in the adult cohort (Figure 3J). Nevertheless, this degree of myeloid-biased differentiation was small with minimal changes in the frequencies of T cells and B cells up to 52 weeks of age (Figure 3K–3L). These mutants had neither anemia nor leukocytosis albeit they also acquired thrombocytopenia at an early age (Figure 3G–3I).

**Figure 4:**
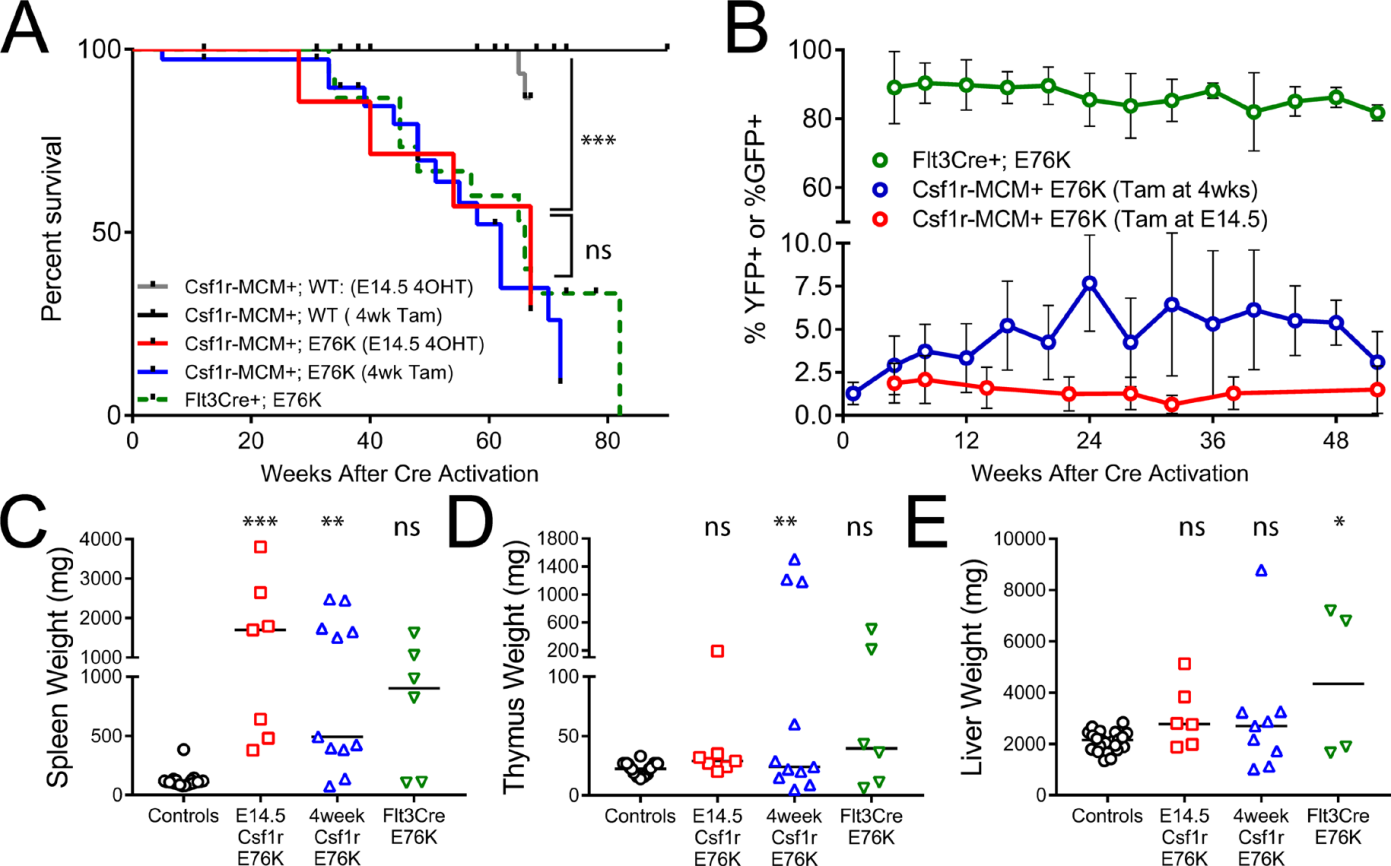
Survival analysis of Ptpn11E76K cohorts (**A**) Kaplan–Meier analysis of survival among Ptpn11E76K cohorts driven by Flt3Cre, Csf1r-MCM activated at 4 weeks of age, and Csf1r-MCM activated at E14.5. (**B**) Comparison of the frequency of YFP or GFP expression in peripheral blood leukocytes in the indicated cohorts. (**C–E**) comparison of tissue weights of moribund animals or littermates analyzed at the end of the study. *P*-values represent *t*-tests comparing each mutant group to the control animals.

Despite their indolent progression of MPN, Csf1r-MCM+;E76K animals from both the fetal and adult cohorts had diminished survival compared to their controls (Figure 4A). Mutants from the fetal cohort had a median survival of 67 weeks after tamoxifen exposure, whereas mutants from the adult cohort had a median survival of 62 weeks. Both Csf1r-MCM+;E76K mutant cohorts had splenomegaly and a small number of animals died with enlarged thymuses (Figure 4C–4E). Interestingly, the survival of the Csf1r-MCM+;E76K cohorts was not different from that of Flt3Cre+;E76K mutants (Figure 4A). This was surprising given that the proportion of cells with active Cre recombinase activity, as measured by the expression of either YFP or GFP, was at least 10-fold greater in Flt3Cre+ animals compared with Csf1r-MCM+ animals (Figure 4B). This result suggested that either i) the morbidity from disease was independent of the mutant allele fraction or ii) that there was an unpredictable relationship between the presence of the fluorescent marker and the expression of Ptpn11E76K.

**Figure 5:**
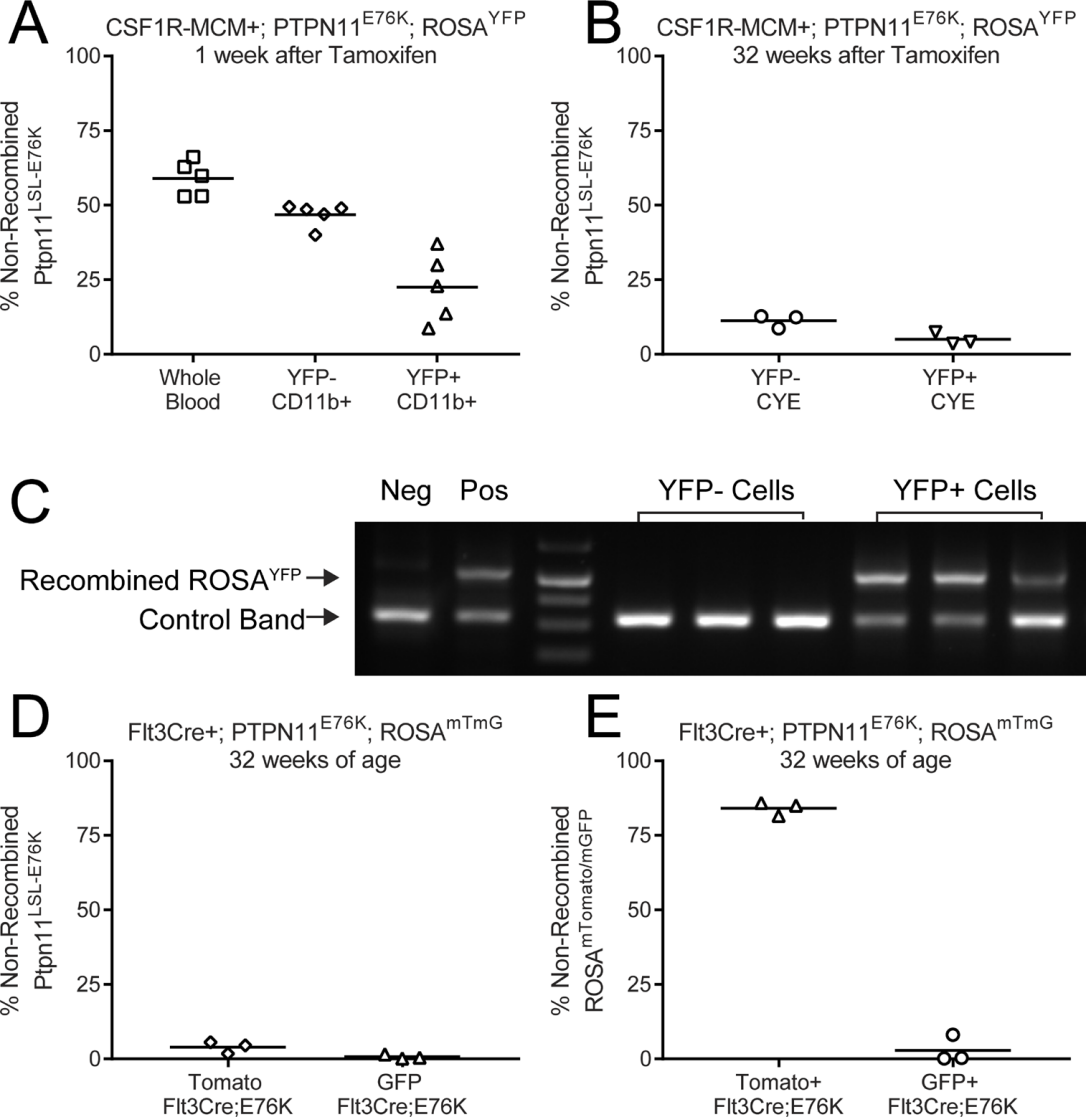
Differential Cre activity in Rosa26 and Ptpn11 loci The frequency of non-recombined Ptpn11LSL-E76K alleles was measured by qPCR in sorted leukocyte subsets. (**A**) In Csf1r-MCM+ Ptpn11E76K peripheral blood leukocytes 1 week after tamoxifen injection. (**B**) In Csf1r-MCM+ Ptpn11E76K peripheral blood leukocytes 32 week after tamoxifen injection. (**C**) PCR analysis of recombination of the Rosa26 locus in leukocyte samples shown in panel A. (**D**) Analysis of sorted GFP+ and Tomato+ peripheral blood CD11b+ Gr1+ neutrophils. (**E**) qPCR analysis of recombination at Rosa26 locus in samples shown in (D).

We therefore proceeded to directly measure the frequency of cells that had recombined the Ptpn11^LSL-E76K^ locus. We designed qPCR primers that would amplify the loxP-flanked DNA sequence that must be excised to permit transcription of mutant Ptpn11. We sorted YFP+ peripheral blood cells from Csf1r-MCM+;E76K animals 1 week after tamoxifen treatment. 22% of YFP+ myeloid cells retained the non-recombined Ptpn11 LSL-E76K allele, indicating that YFP had a positive predictor value (PPV) of 78% for mutant allele expression (Figure 5A). In contrast, the negative predictive value of YFP expression for Ptpn11E76K expression was only 47%, as measured by the proportion of YFP- cells that had recombined the mutant locus (Figure 5A). 32 weeks after tamoxifen, the PPV of YFP expression for Ptpn11 recombination improved to 96%, albeit the NPV fell to 12% (Figure 5B). We repeated this analysis using sorted GFP+ and Tomato+ cells from 32 week-old Flt3Cre+;E76K mice (Figure 5D). The PPV of GFP for mutant allele recombination was 99% but the NPV was only 5% (Figure 5D). We confirmed that fluorescence expression was an excellent predictor of recombination at the Rosa26 locus, indicating that YFP and GFP expression were not being silenced in our animals (Figure 5C, 5E). These results demonstrate that the frequency of Ptpn11E76K-expressing leukocytes is much greater than that predicted by our fluorescent markers. Additionally, these results show that Csf1r-MCM+;E76K animals have only marginally fewer mutant-expressing leukocytes when compared to Flt3Cre+;E76K animals.

**Figure 6:**
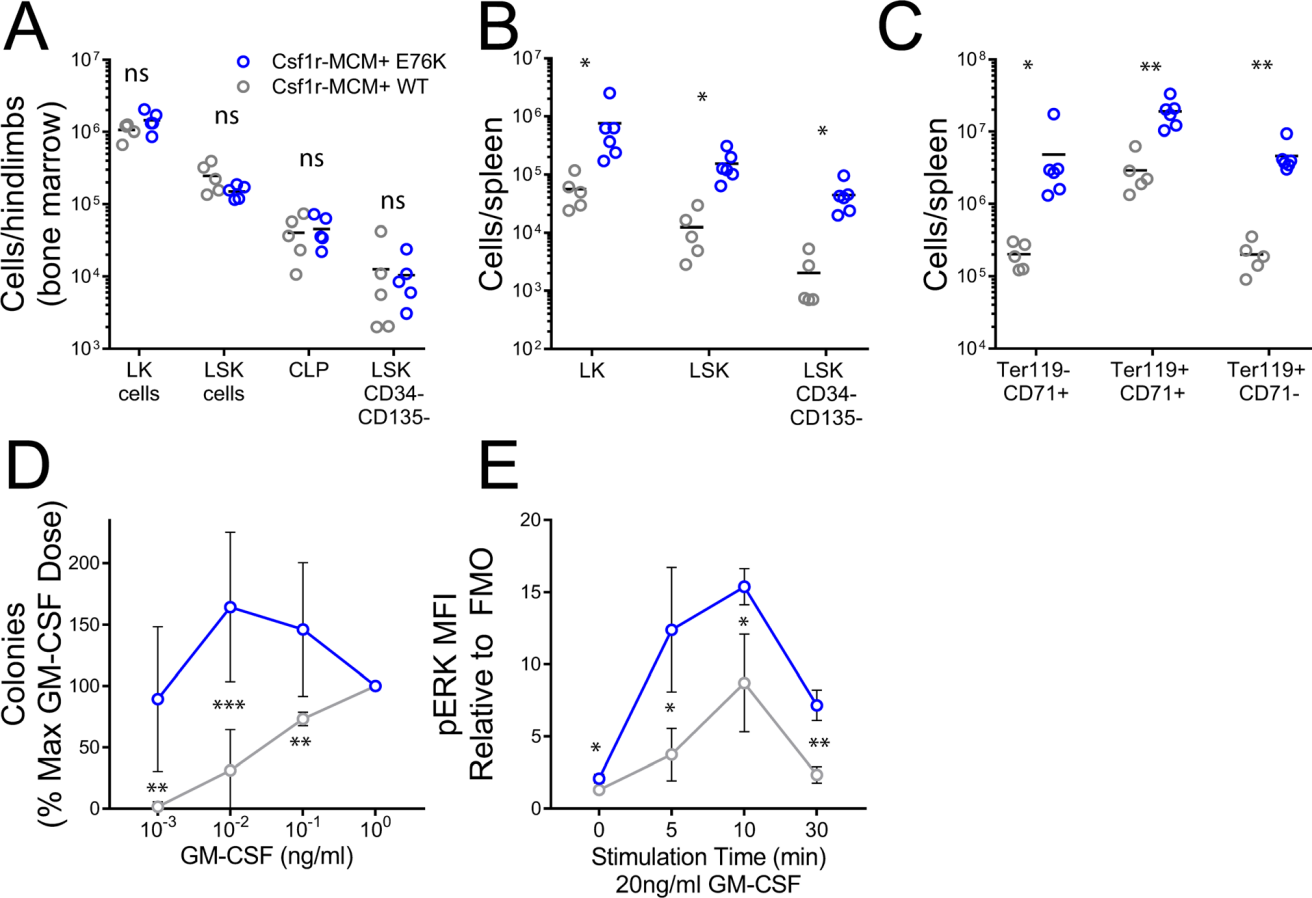
Progenitor analysis in Csf1r-MCM+ Ptpn11E76K All animals were exposed to tamoxifen at 4 weeks of age and analyzed 32 weeks later. (**A**) Abundance of BM progenitors. (**B**) Abundance of splenic progenitors. (**C**) Abundance of erythroid progenitors in the spleen. (**D**) Methylcellulose colony assays were performed at indicated doses of GM-CSF. Colonies were scored on day 7 (*n =* 6 biologic replicates/group). (**E**) p-ERK expression in BM-derived macrophages following stimulation with GM-CSF, as measured by intracellular flow cytometry (*n =* 3 biologic replicates/group). Fluoresence minus one (FMO) controls were used to define background fluorescence absence of p-ERK staining. All *p*-values represent *t*-tests comparing mutants and controls.

We proceeded to analyze progenitor populations in Csf1r-MCM+;E76K animals from the adult cohort to further evaluate their cause of death. 32 weeks after tamoxifen injection, mutants had equivalent numbers of BM progenitors compared to controls (Figure 6A). However, there was an increase in the number of splenic LK cells, LSK cells, and HSCs in mutants compared to controls (Figure 6B). The most pronounced difference was in the frequency of CD45– erythroid progenitors—pro-erythroblasts (CD71+ Ter119–), basophilic erythroblasts (CD71+ Ter119+), and late erythroblasts (CD71low/dim Ter119+) (Figure 6C). We proceeded to confirm whether progenitors from our model had two pathognomonic features of JMML: growth hypersensitivity to GM-CSF and hyperactive Ras-Erk signaling. We plated BM cells at increasing doses of GM-CSF and observed that mutant progenitors gave rise to more colonies at low cytokine doses compared to controls (Figure 6D). Next, we generated BM-derived macrophages from mutants and controls and measured phosphorylation (p-) of ERK following stimulation with 20 ng/ml GM-CSF. Mutant macrophages had markedly greater p-ERK expression than control macrophages at baseline and up to 30min following cytokine stimulation (Figure 6E). As expected given the comparable frequency of Ptpn11E76K expression, no difference in colony formation and p-ERK expression was observed between YFP+ and YFP- cells (Supplementary Figures 5–6).

**Figure 7:**
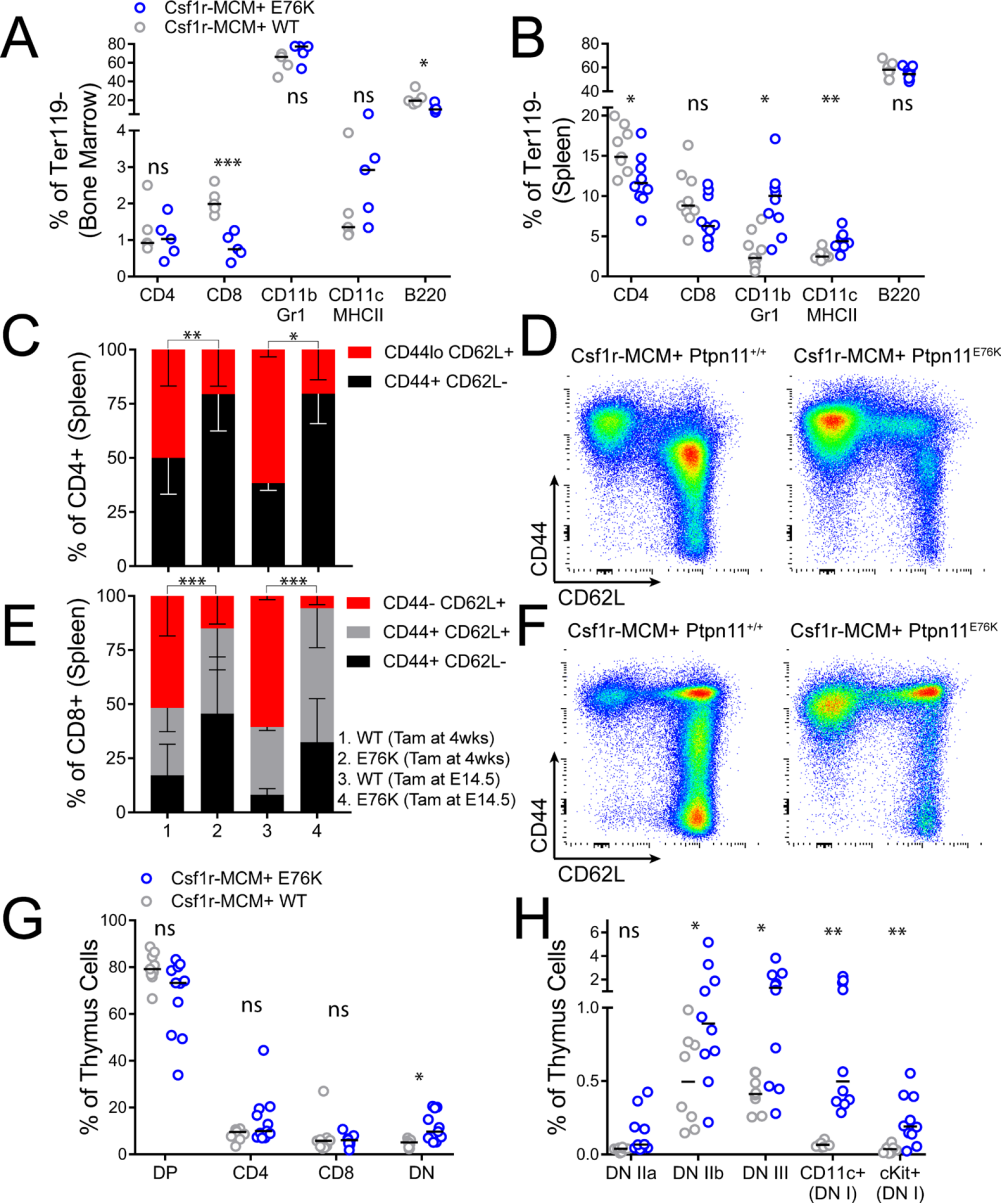
T-lymphocyte analysis in Csf1r-MCM+ Ptpn11E76K All animals were exposed to tamoxifen at 4 weeks of age and analyzed 32 weeks later, unless indicated. (**A, B**) Frequency of myeloid cells, dendritic cells, B-cells, and T- cells in the BM and spleen of mutants and littermates. (**C, D**) Frequency of naïve and effector CD4+ cell subsets with representative gating. (**E, F**) Frequency of naïve and effector CD8+ cell subsets with representative gating. (**G, H**) Distribution of T-lymphocyte progenitors in the thymuses of mutants and littermates. DP, CD4 CD8 double positive. DN, double negative. *P*-values in panels C and E represent two-tailed *t*-tests comparing the frequency of naïve T cells.

Finally, we proceeded to characterize the distribution of leukocytes in tissues of our Csf1r-MCM+ adult cohort. Mutant animals had fewer CD8+ BM T-cells and fewer CD4+ splenic T-cells compared to controls (Figure 7A, 7B). They also had modest expansion of splenic CD11b+ Gr1+ neutrophils and CD11c+ MHCII+ dendritic cells (Figure 7B, Supplementary Figure 7). We proceeded to further evaluate T-cell subsets in mutant animals by comparing the relative frequency of naïve vs. memory T-lymphocytes. Mutant animals in both the adult and the fetal cohorts had markedly diminished CD44-/low CD62L+ naïve cells among both CD4+ and CD8+ T-cells (Figure 7C–7F). We reasoned that this depletion of naïve T-cells may prompt an increase in the number of T cell progenitors. Indeed, whereas the number of CLPs in the BM of mutant animals was not increased (Figure 5A), there was an marked increase in the frequency of early T cell progenitors in the thymus (Lin- cKit+) as well as committed CD4- CD8- CD25+ progenitors, DN IIb progenitors (CD44+ cKit-), and DN III progenitors (CD44– cKit-) (Figure 7G, 7H, Supplementary Figure 8).

## DISCUSSION

We present the first hematopoietic-restricted animal models of Ptpn11E76K that avoid myeloablation. We demonstrate that Flt3Cre+;E76K and Csf1r-MCM+;E76K mice have a markedly prolonged survival compared with previous conditional models that expressed this mutation with non-hematopoietic-restricted Cre strains. We thereby show that Ptpn11E76K-expressing hematopoietic progenitors in mice give rise to an indolent disease that has delayed onset of anemia, minimal monocytosis, and inconsistent leukocytosis. Rather, the dominant pathology of Ptpn11E76K is early-onset thrombocytopenia with a marked increase in the frequency of extramedullary erythropoiesis.

Given that JMML’s origin is intimately associated with fetal blood development, we sought to compare the contribution of fetal and adult hematopoietic progenitors to disease. We devised an inducible Cre-loxP strategy whereby the timing of tamoxifen administration would determine the temporal expression of Ptpn11E76K. Previous studies have demonstrated that the transition from fetal to adult hematopoiesis in the mouse is complete by 4 weeks of age [28]. The corresponding transition in humans was measured to occur at 2 years of age [29, 30]. We therefore chose to induce Cre activity at our model at E14.5 or at 4 weeks of age to target fetal and adult progenitors, respectively. We chose the Csf1r-MCM strain because this promoter is active in hematopoietic progenitors, including HSCs [31]. Our results confirm that tamoxifen injection at E14.5 and 4 weeks of age can durably target a subset of hematopoietic progenitors, as measured by the expression of YFP. We therefore proceeded to generate fetal and adult cohorts of Csf1r-MCM+;E76K animals and compare their disease progression with that of Flt3Cre+;E76K animals.

Mutants in our fetal cohort developed monocytosis more rapidly than mutants in the adult phase, and this myeloid-biased differentiation continued until 52 weeks of age. This is consistent with the greater proliferative and myeloid-colony forming potential of fetal progenitors compared with adult progenitors [23, 32]. These findings support the hypothesis that the fetal hematopoietic microenvironment is more prone to the development of JMML-like myeloproliferative features. Nevertheless, the overall burden of disease was minimal; both fetal and adult Csf1r-MCM+;E76K cohorts had delayed onset of anemia, minimal monocytosis, and lacked leukocytosis. This suggests that the morbidity elicited by hematopoietic-restricted Ptpn11E76K is considerably less than that of previous models, such as Mx1Cre+;Ptpn11E76K, wherein oncogene expression was not limited to blood cells. As such, our findings are consistent with the recent findings that Ptpn11E76K-expressing BM stromal populations exacerbate MPN [19]. We acknowledge that we cannot exclude the possibility that altered housing conditions or animal microbiomes contribute to differences between our E76K model and those reported previously. Nevertheless, our results strongly advocate for future studies of MPN and leukemia to use techniques that can distinguish hematopoietic from non-hematopoietic burdens of disease.

Fetal and adult Csf1r-MCM+;E76K cohorts had similar survival following exposure to tamoxifen. This suggests that the early onset of monocytosis in the fetal cohort was not a major determinant of morbidity. Rather, thrombocytopenia and expanded extramedullary erythropoiesis were the most pronounced phenotypes in our adult cohort mutants and likely had the greatest contribution to mortality. These findings are consistent with earlier studies that have noted expanded extramedullary hematopoiesis and defective erythropoiesis in JMML animal models [9, 11, 33]. One potential mechanism for this mobilization is a pro-inflammatory BM niche that provokes extramedullary seeding of progenitors. It is notable, however, that our models did not show the same degree of monocytosis as is observed in JMML patients. One potential explanation are the sub-clonal secondary mutations in Ras signaling mediators that confer a worse prognosis among a subset of JMML patients [1–3]. In contrast, our models expressed a single oncogenic mutation and it is likely that the presence of additional oncogenes in our mice would evoke a more rapidly-progressing JMML-like MPN.

We expected Flt3Cre and Csf1r-MCM models of Ptpn11E76K to produce markedly different MPN manifestations. Flt3Cre is constitutively active, indicating that the mutation will be expressed *de novo* each time an HSC differentiates through a Flt3+ intermediate. Furthermore, Flt3Cre is highly efficient and targets approximately 90% of leukocytes. In contrast, Csf1r-MCM is only active in Csf1r+ cells in the presence of tamoxifen. As such, mutagenesis should occur during a single brief interval of time in a small subset of cells. We reasoned that this strategy would more accurately represent the clonal nature of leukemogenesis whereby a handful of oncogene-expressing cells expand amidst neighboring WT cells.

Our lineage trace of Csf1r-MCM+;Rosa26YFP mice confirmed that this Cre could target a small subset of hematopoietic progenitors (Figure 2). Furthermore, we observed an expansion of mutant YFP+ cells in our adult Csf1r-MCM+;E76K cohort to 24 weeks (Figure 4B). However, the frequency of YFP+ cells plateaued at 8% and did not increase further up to at least 52 weeks of age. This plateau prompted us to evaluate the faithfulness of our fluorescent reporters for oncogene expression. We determined that the majority of YFP- cells had inappropriately recombined their Ptpn11 LSL-E76K allele. As a result, there was no correlation between fluorescence and oncogene expression. Our results further showed that Flt3Cre and Csf1r-MCM models had nearly-identical proportions of Ptpn11E76K-expressing blood cells. As such, we could not evaluate whether near-clonal expression of Ptpn11E76K is sufficient to give rise to MPN in a mouse model. We do demonstrate, however, that both Csf1r-MCM and Flt3Cre have different recombination efficiencies at different loci and that oncogene expression cannot be inferred from fluorescence-based lineage trace studies.

The role of T cells in our model is intriguing. We noted that Flt3Cre+;E76K and Csf1r-MCM+; E76K models had decreased frequency of T-lymphocytes. CD4+ cells were preferentially reduced compared with CD8+ cells. Furthermore, the distribution of CD4+ and CD8+ cells favored effector cells (CD44hi CD62L neg/lo) whereas there was a paucity of naïve cells (CD44 low/neg CD62L+). This suggests that T-cells are being activated by antigen presenting cells, leading to their exhaustion and depletion. This is supported by our findings in the thymus, where we observed a relative increase of CD4- CD8- progenitors, including cKit+ CD44+ CD25- early thymic progenitors. This suggests that the thymus of mutant animals may undergo greater turnover, which may predispose to tumorigenesis. Indeed, we observed that 25% of mutant animals in our studies died with large thymuses, which is suggestive of T-ALL.

Our T-cell findings have notable parallels in JMML patients. Occasionally, these children acquire T-ALL either concurrently with their MPN or after the resolution of their myeloid disease [18, 34–36]. Our observation of decreased naïve peripheral T cells and increased thymic precursors may provide a mechanistic explanation for this association. Admittedly, it is rare for a patient to have both JMML and T-ALL. However, this low rate is not surprising given that T cells in most JMML patients do not express the disease-initiating mutation [1, 37]. This lack of expression may be the result of a block in T cell maturation among mutation-expressing progenitors. This is supported by case reports that have suggested JMML patients have decreased T cell frequencies compared with healthy controls [38, 39]. Alternatively, the kinetics of T cell development in the thymus may delay the presence of mutated T cells in the periphery as compared with mutated myeloid progeny. It will be informative for future studies to definitively measure the frequency of T cells in JMML patients and to assess the relative contribution of their CD4+ and CD8+ cells as well as their naïve and effector subsets.

In summary, we present the first hematopoietic-restricted model of Ptpn11E76K expression that does not rely on transplantation. We demonstrate that *in utero* mutations in fetal progenitors result in more rapid monocytosis compared with mutations in adult progenitors. Nevertheless, our models had indolent MPN progression as measured by anemia, leukocytosis, and monocytosis. Instead, our models developed splenomegaly due to pronounced extramedullary erythropoiesis and thrombocytopenia. Finally, we demonstrate mutant mice to have a paucity of T lymphocytes, most notably a deficit of CD4+ and memory T cells. Our findings reveal the temporal progression of MPN in models of JMML and suggest that abnormal extramedullary erythropoiesis and T-lymphopoesis may have a significant contribution to morbidity in JMML patients.

## MATERIALS AND METHODS

### Study approval

Animal studies were approved by the IACUC at the Indiana University School of Medicine.

### Mice

C57BL/6J mice were bred in-house. Flt3Cre+;ROSA^mTmG/mTmG^ mice were a kind gift from Dr. Slava Epelman (University of Toronto). LSL-PTPN11^E76K/+^ mice were obtained from Dr. Cheng-Kui Qu (U. Emory, Atlanta, GA). ROSA^YFP/YFP^ mice were obtained from Dr. Anthony Firulli (IUSM, Indianapolis, IN). CSF1R-Mer-Cre-Mer mice (#019098) were purchased from Jackson Labs and were back-crossed onto the C57B6 background for 6 generations. Mice were identified by ear notches or toe clips. Genotyping was performed with conventional PCR using primers listen in the Supplement.

### Timed matings

Male studs (10–26 weeks of age) were housed in separate cages and mated after at least 2–3 days of acclimatization to their cage. In the evening one or two female mice (8–26 weeks of age) were moved to the stud cage. The following morning, successful matings were confirmed by visual inspection of a vaginal plug and assigned a gestational age of E0.5.

### Tamoxifen treatment

To induce recombinase activity *in utero*, pregnant dams were injected i.p. with 75 ug/g 4-hydroxy tamoxifen (Sigma #H6278) along with 37.5 ug/g progesterone (Sigma #P0130). Litters of tamoxifen-treated dams were routinely delivered by C-section on E19.5 and raised by foster females.

### Cell isolations

Blood was collected into EDTA-coated tubes and analyzed using the HemaVet 950FS hematology analyzer (Drew Scientific). Mouse tissues were kept on ice in PBS + 2 mM EDTA. Bone marrow cells were flushed from hindlimb bones. Spleens and thymuses were triturated with glass cover slides. BM stromal populations were isolated by incubating flushed bones in 22 U/ml collagenase type 2 (Worthington Biochemical #LS004177) for 45 min at 37°C.

### Flow cytometry

Cells were stained at a concentration of 1–5 × 10^7^/ml in PBS +2% FBS + 2 mM EDTA. Antibody concentrations were determined experimentally. If required, cells were fixed in 1% PFA (Fisher #50-980-487). For Intracellular phospho-flow, cells were stimulated in an Eppendorf tube with GM-CSF (Peprotech #315-03), fixed in 1% PFA for 15 min, and permeabilized using BD Perm Buffer III (#558050). Stained cells were analyzed using the BD LSR Fortessa, BD FACS CANTO II, or BD Accuri C6. Cell sorting was performed on a BD FACSAria. Post run analysis was performed using FlowJo (Treestar). Percentages in graphs represent each lineage’s frequency among live mononuclear cells, as determined by FSC and SSC gating.

### Methylcellulose progenitor assays

5 × 10^4^ BM mononuclear cells were plated in 1 ml of methylcellulose medium (40% ES-Cult M3120 (Stem Cell Technologies), 30% FBS (HyClone), 220 U/ml Pen/Strep, 1.2 mM L-Glutamine, and 440 nM β-Mercaptoethanol) and defined concentration of murine GM-CSF (Peprotech #315-03). Colonies were counted 7 days after plating.

### qPCR

Genomic DNA was isolated from cells using the Qiagen DNeasy Blood & Tissue Kit (#69504). Samples were prepared using FastStart Universal SYBR Green Master (Roche #04913850001) and were run on a 7500 Real-Time PCR System (Applied Biosystems). Primer sequences are listed in the Supplement.

### Histology

Mouse femurs were fixed in 4% phosphate buffered formaldehyde overnight, stored in 70% ethanol, demineralized with EDTA, paraffin embedded, sectioned to 5 µm, and stained using hematoxylin and eosin.

### Statistics

Statistical analysis was performed using GraphPad Prism 7.0. *P* values comparing mutant and littermate groups were calculated using two-tailed Student’s *t* tests, one-way ANOVA, Mantel Cox log rank tests, or chi-squared test. *P* values < 0.05 were considered significant. All error bars represent SD. *P*-value notations: ns *p* > 0.05, ^*^p < 0.05, ^**^P < 0.01, ^***^p < 0.001.

## SUPPLEMENTARY MATERIALS FIGURES AND TABLES



## Abbreviations

4OHT: 4 hydroxy-tamoxifen
CMoP: common monocyte progenitor
CMP: common myeloid progenitor
Csf1r: colony stimulating factor 1 receptor
DN: double negative (CD4– CD8–)
DP: double positive (CD4+ CD8+)
EC: endothelial cells
GFP: green fluorescent protein
GM-CSF: granulocyte-macrophage colony stimulating factor
GMP: granulocyte-macrophage progenitor
HSC: hematopoietic stem cell
JMML: Juvenile Myelomonocytic Leukemia (JMML)
LK: Lin– cKit+ cells
LSK: Lin- cKit+ Sca1+ cells
MCM: Mutated estrogen receptor – Cre – Mutated estrogen receptor
MDP: myeloid-dendritic progenitor
MEP: megakaryocyte-erythrocyte progenitor
MPN: myeloproliferative neoplasm
MSPC: mesenchymal stem/progenitor cell
NPV: negative predictive value
OB: osteoblast
PPV: positive predictive value
Ptpn11: protein tyrosine phosphatase 11
YFP: yellow fluorescent protein
