# Supplementary material for: Hematopoietic-restricted Ptpn11E76K reveals indolent MPN progression in mice
Source: Oncotarget. 2018 Apr 24;9(31):21831–43. doi: 10.18632/oncotarget.25073 (PMC5955130; doi:10.18632/oncotarget.25073)
Supplement: Supplementary file 1 [file oncotarget-09-21831-s001.pdf]

Hematopoietic-restricted Ptpn11E76K reveals indolent MPN progression in mice

SUPPLEMENTARY MATERIALS

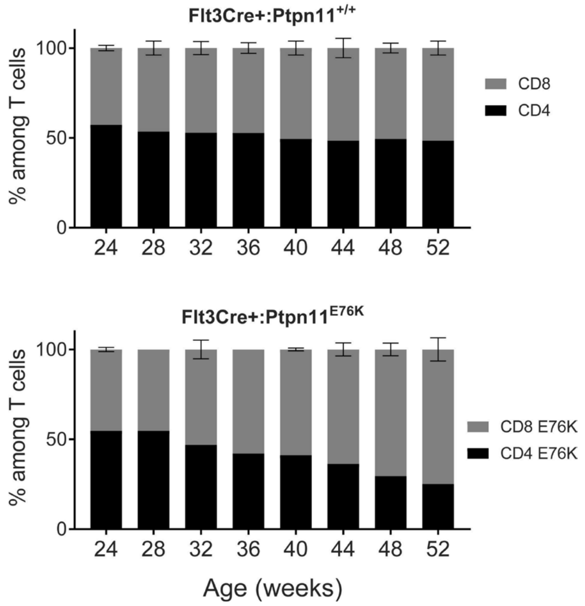

Supplementary Figure 1: Flt3Cre+;E76K show progressive loss of CD4+ T-cells. Flow cytometric analysis of peripheral blood CD4+ and CD8+ T cells from Flt3Cre+;E76K mutant animals and littermate controls. Related to Figure 1F.

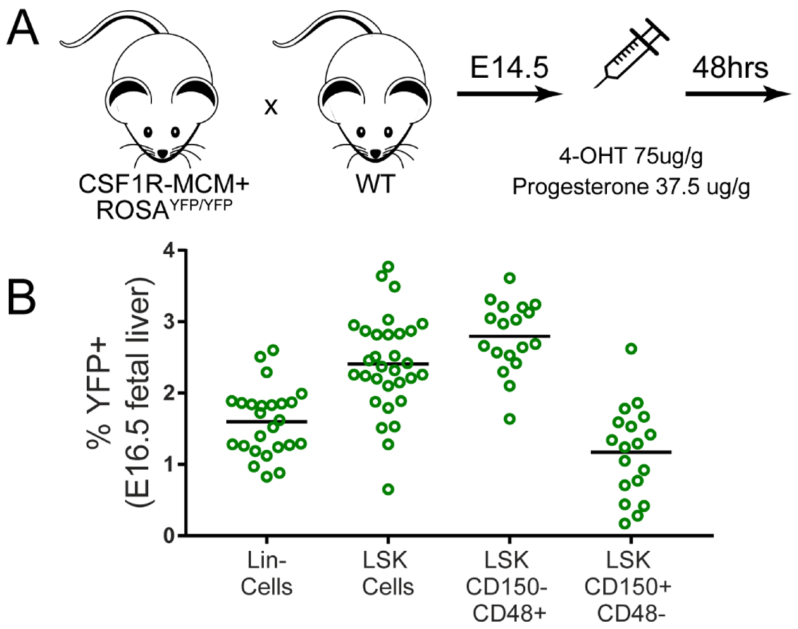

Supplementary Figure 2: Csf1r MER-Cre-MER activity in fetal hematopoietic progenitors. (**A**) Csf1r-MCM+;Rosa26$^{YFP/YFP}$ animals studs were mated with C57B6 females. On E14.5 a 75 ug/g dose of 4-hydroxytamoxifen and 37.5 ug/g progesterone was injected into pregnant dams. (**B**) Frequency of hematopoietic progenitors in fetal livers on E16.5.

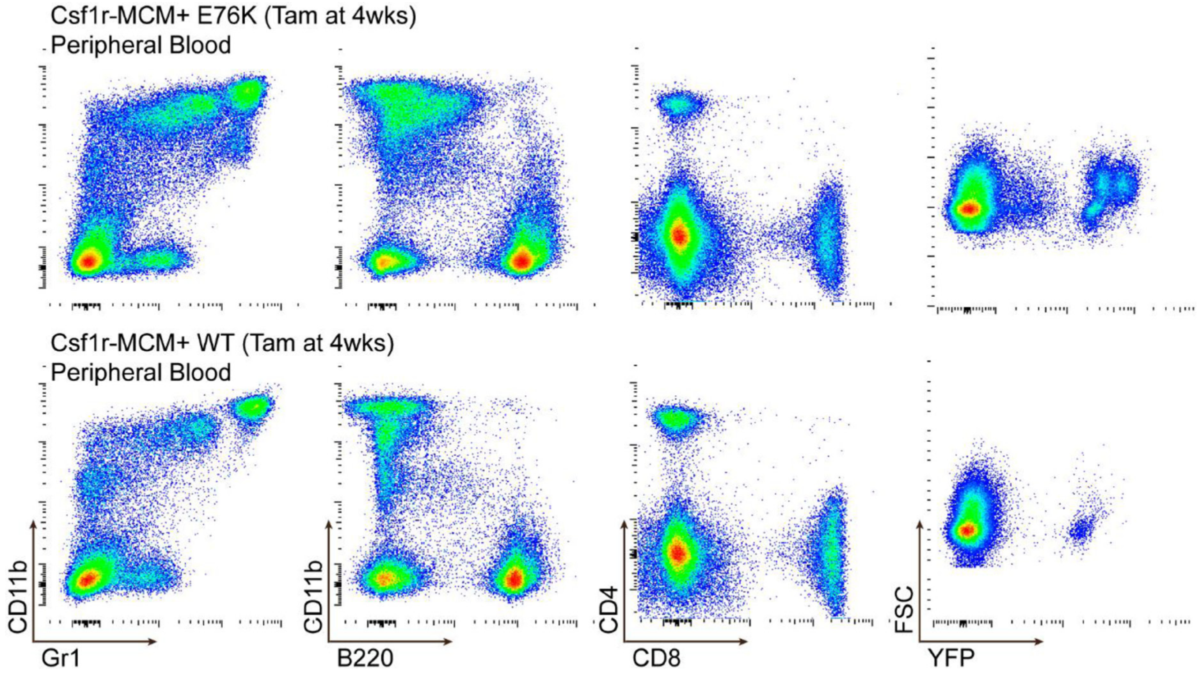

Supplementary Figure 3: Representative flow cytometric gating of peripheral blood in Csf1r-MCM+;E76K animals and controls.

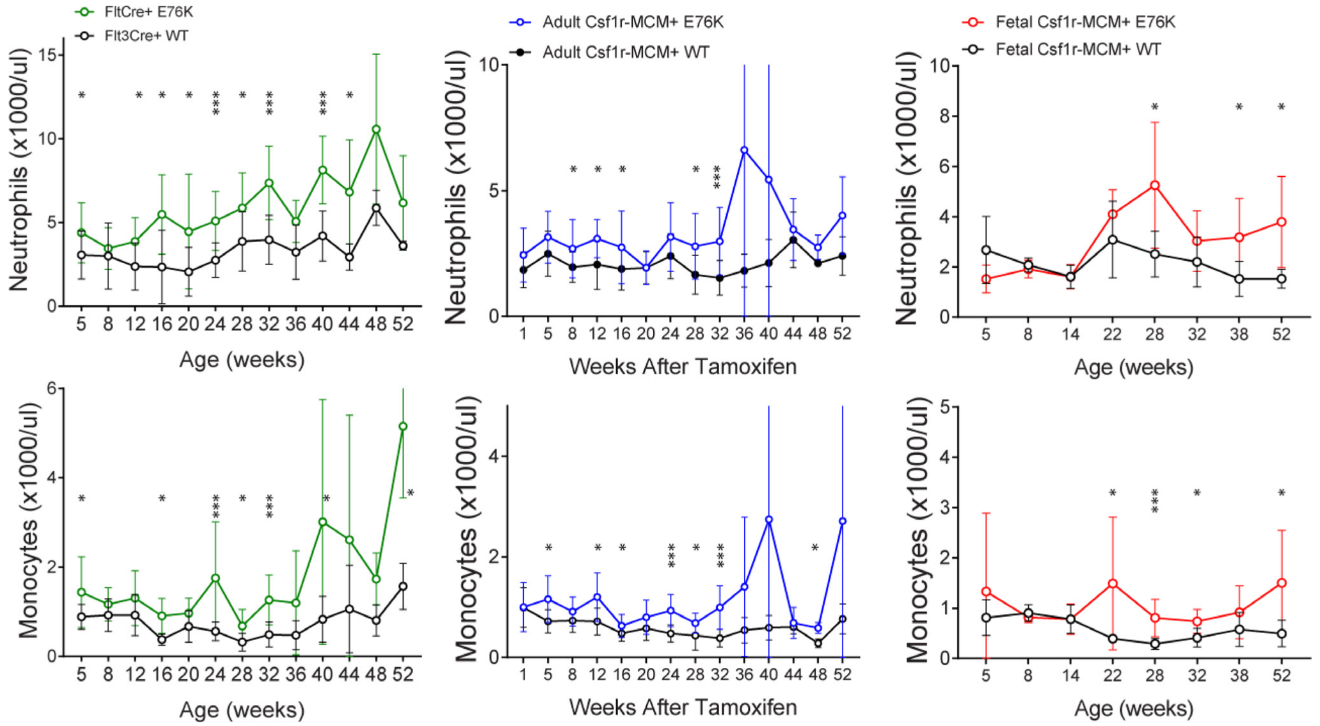

Supplementary Figure 4: Absolute monocyte and neutrophil counts in animal models as measured by an automated hematology analyzer.

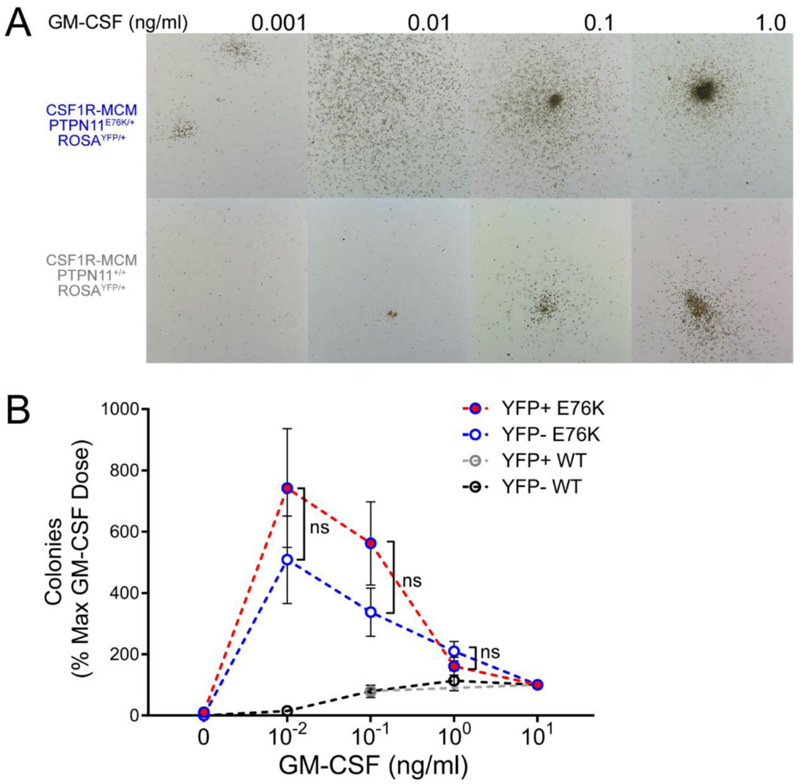

Supplementary Figure 5: GM-CSF hypersensitivity among YFP+ and YFP- progenitors in Csf1r-MCM+;E76K animals. (**A**) Representative colonies obtained 7 days after plating BM cells from Csf1r-MCM+;E76K mice and littermate controls. (**B**) YFP+ and YFP- Lin- cKit+ CD34+ progenitors were sorted from BM of 32 week of Csf1r-MCM+;E76K animals and littermate controls and plated in methylcellulose colony forming assays at indicated doses of GM-CSF.

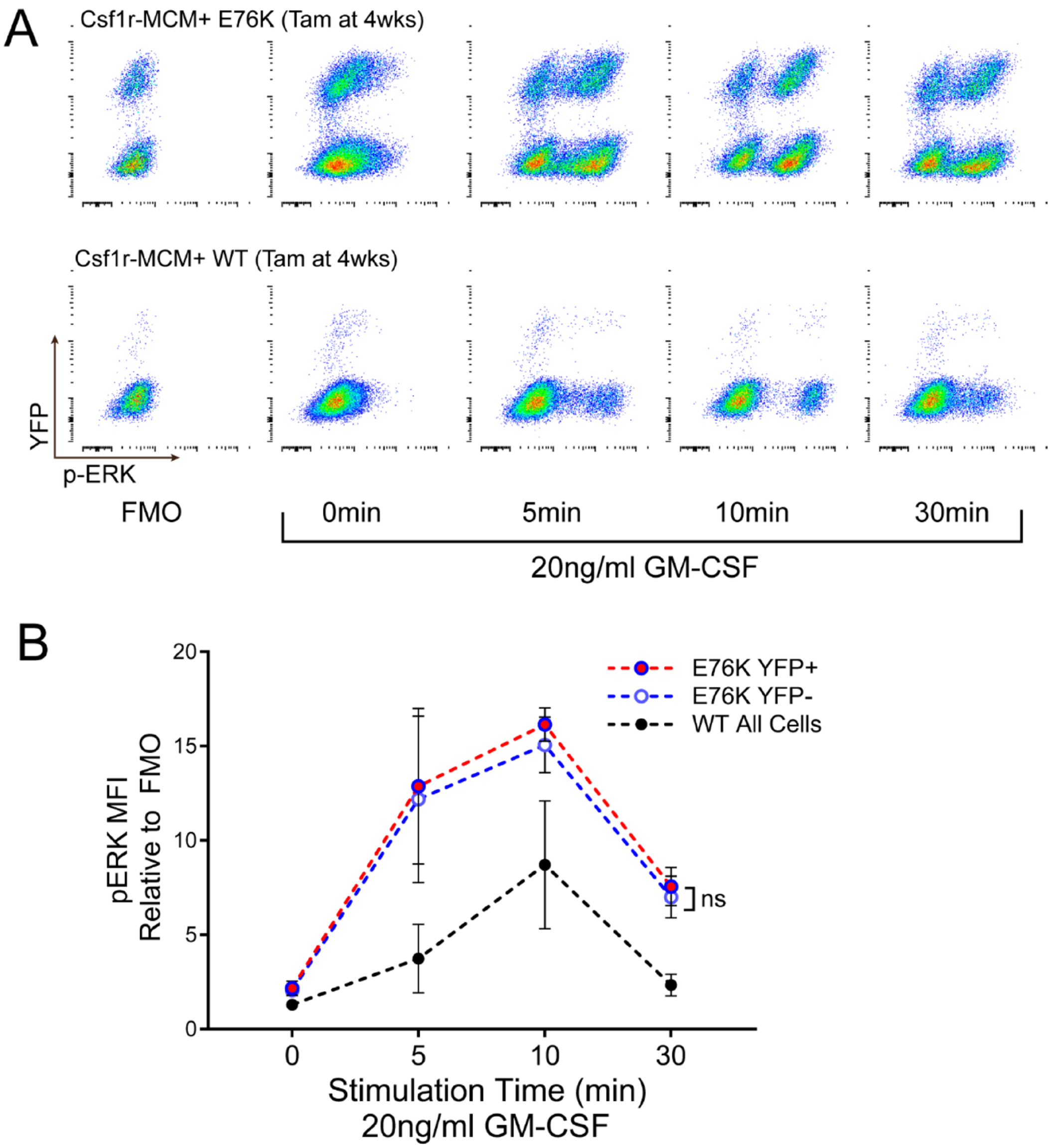

Supplementary Figure 6: Ras-Erk Hyperactivity in Csf1r-MCM+;E76K animals. BM cells from mutant and control animals were *ex vivo* differentiated into macrophages via 7 day culture in 10 ng/ml M-CSF. Macrophages were stimulated with indicated doses of GM-CSF and p-ERK expression was measured using flow cytometry. (**A**) Representative flow cytometric gating of stimulated cells. (**B**) Mean fluorescence intensity of p-ERK expression among YFP+ mutant cells, YFP– mutant cells, and WT cells following stimulation with GM-CSF.

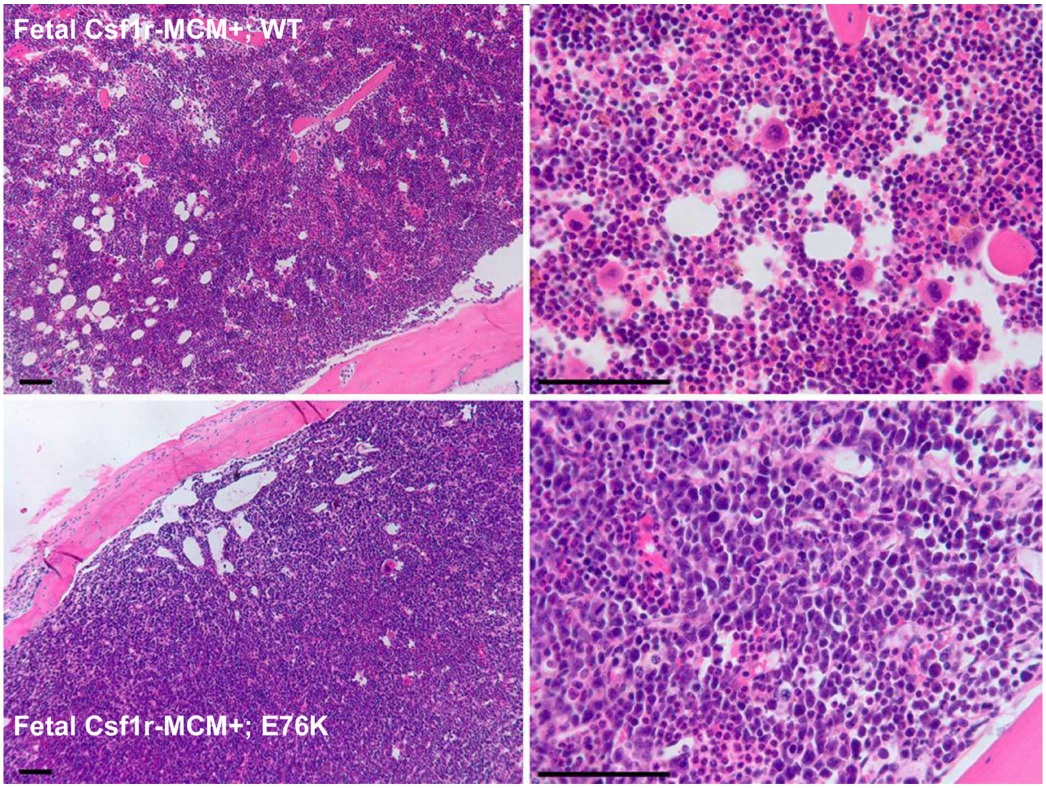

Supplementary Figure 7: Representative hematoxylin and eosin sections of femurs from fetal Csf1r-MCM+;E76K animals (top row) and fetal Csf1r-MCM+; WT controls (bottom row). Scale bars represent 100 µm.

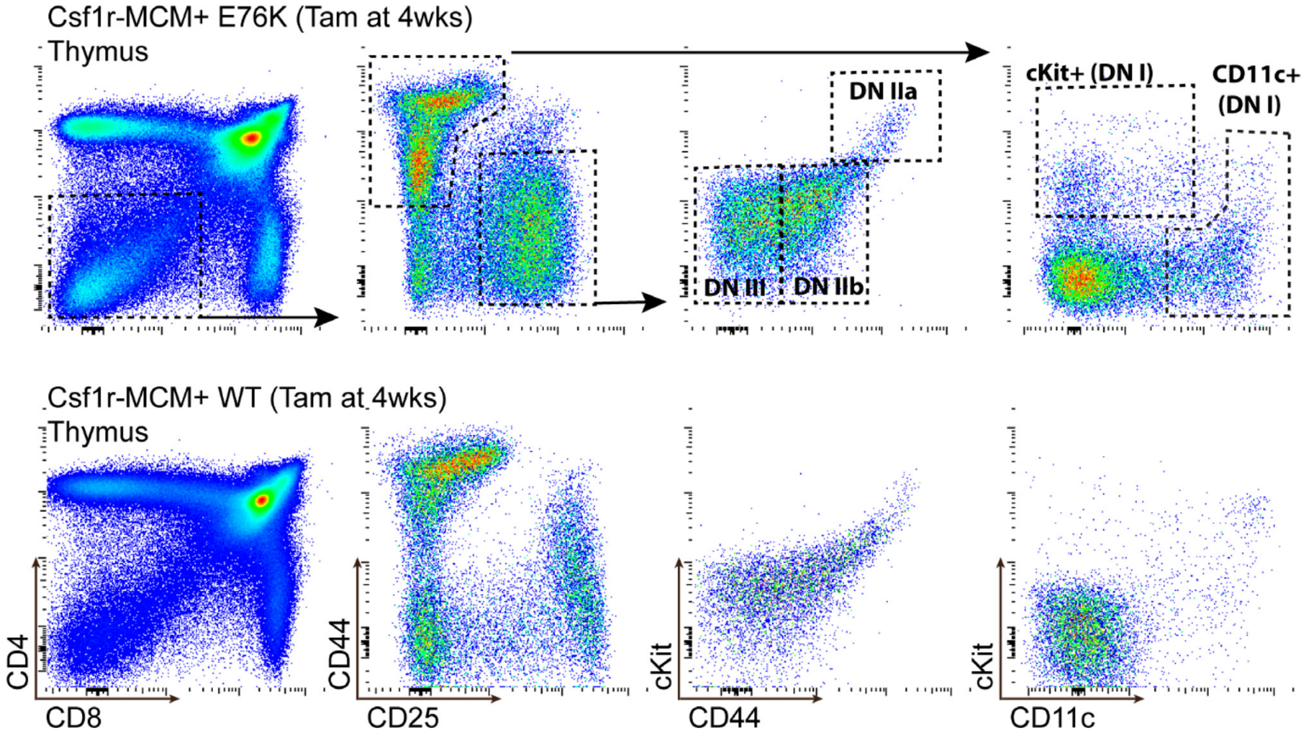

Supplementary Figure 8: Representative flow cytometric gating of T cell progenitors in the thymus. DP, CD4 CD8 double positive. DN, double negative, Tam, tamoxifen.

Supplementary Table 1: List of oligonucleotides used for conventional and quantitative PCR

Reaction	Primers	Interpretation
Sly/Xlr for Flt3Cre genotyping	GATGATTTGAGTGGAAATGTGAGGTA	Male = 280bp (Flt3Cre+)
	CTTATGTTTATAGGCATGCACCATGTA	Female = 480bp + 660bp + 685bp
Csf1r-MCM genotyping	CTA GGC CAC AGA ATT GAA AGA TCT	Csf1r-MCM+ 236bp
	GTA GGT GGA AAT TCT AGC ATC ATC C	Internal control = 324bp
	AGA TGC CAG GAC ATC AGG AAC CTG	
	ATC AGC CAC ACC AGA CAC AGA GAT C	
Ptpn11E76K genotyping	TGGGAAGACAATAGCAGGCA	E76K allele = 349bp
	CCCACTCACCTTGTCATGTA	
ROSA26 YFP: genotyping	AAAGTCGCTCTGAGTTGTTAT	Recombined: 550bp
	AAGACCGCGAAGAGTTTGTC	YFP allele = 320bp
	AGCTC CTCGCCCTTGCTCACCATG	
Ptpn11E76K: qPCR recombination	GCTTCTGAGGCGGAAAGAAC	
	TGGAGAAATGGCTTGGATCCC	
ROSA26 mTmG: qPCR recombination	GTGAACCTCTTCGAGGGACC	
	GTCACCTTCAGCTTGGCGG	
POLR2A: qPCR recombination. Control.	TTTTGCGCTGTGTCTGCTTC	
	AATTGGAGCTGTTCCCTTGC	

Supplementary Table 2: List of antibody clones used for flow cytometry

Antibody	Clone
B220	RA3-6B2
CD117	2B8
CD11b	M1/70
CD11c	N418
CD135	A2F10
CD140a	APA5
CD150	TC15-12F12.2
CD16/32	93
CD25	PC61
CD3	145-2C11
CD31	390
CD34	HM34
CD4	GK1.**5**
CD44	IM7
CD45	104
CD48	HM48-1
CD51	RMV-7
CD71	RI7217
CD8	53-6.**7**
Gr1	RB6-8C5
MHCII	M5/114.15.2
p44/42MAPK(Erk1/2)	L34F12
Sca1	A2F10
Ter119	TER-119
